# A mixed method evaluation of knowledge, attitude and practice on dengue fever among Lalitpur Metropolitan City residents: a cross-sectional investigation

**DOI:** 10.1186/s12879-024-10025-8

**Published:** 2024-10-08

**Authors:** Sushmita Ghimire, Shraddha Pangeni

**Affiliations:** 1 Department of Public Health, Asian College for Advance Studies, Lalitpur, Nepal; 2 Center for Health and Disease Studies Nepal, Kathmandu, Nepal; 3 Medical Officer, Freelancer at the time of study, Kathmandu, Nepal

**Keywords:** Knowledge, Attitude, Practice, Dengue, Aedes, Epidemic

## Abstract

**Background:**

Dengue poses a significant global public health challenge, including in Nepal. Understanding community’s knowledge, attitudes, and behaviors concerning dengue fever is imperative to developing effective prevention and control strategies. This study aimed to assess the knowledge, attitude, and practices related to dengue fever among residents of Lalitpur Metropolitan City.

**Methods:**

A descriptive cross-sectional household study was conducted using a mixed-method approach, which included quantitatively studying 636 individuals and conducting 20 qualitative interviews. The data was collected between April 2023 and June 2023. The multistage cluster sampling method was applied for household selection during the quantitative study and a purposive judgmental sampling method was used to identify participants for the qualitative interviews. Face-to-face interviews were conducted using a structured questionnaire for the quantitative study and an interview guide for the qualitative study. Quantitative data were analysed using logistic regression in STATA version 13, and thematic analysis was applied to the qualitative data. The findings were validated through triangulation of results from both the qualitative and quantitative study.

**Results:**

Regarding knowledge, 64.94% (*n* = 413/636) reported being informed about dengue fever. In terms of attitude, a substantial majority, 91.51% (*n* = 582/636), expressed a positive attitudes toward dengue fever, indicating a favorable perception and knowledge of its significance. Concerning practice, 49.84% (*n* = 317/636) of respondents reported actively engaging in dengue fever prevention measures. The variables gender, previous history of dengue fever and residency were the determinants of dengue fever knowledge. Additionally, gender, residency, and attitude were predictors of preventive practices concerning dengue fever.

**Conclusion:**

Our study revealed that while the community demonstrated good knowledge of dengue fever and positive attitudes toward prevention, their preventive practices were inconsistent, indicating a gap between knowledge and action. A positive attitude was linked to better adherence to preventive measures. To address this gap, it is crucial to promote a positive attitude toward dengue prevention through initiatives like education efforts and social mobilization programs. Implementing Social and Behavior Change Communication (SBCC) programs focused on dengue prevention and control measures can help bridge this knowledge-action gap.

## Introduction

Dengue fever is a significant concern for public health worldwide, particularly in tropical and subtropical regions, as well as countries with limited health resources. Although it has the potential to be fatal, it is preventable [1]. Despite advances in developing and testing vaccines for Dengue Virus (DENV) infection, there is currently no vaccine available for purchase, and there is no specific treatment for Dengue fever (DF) [2]. Since effective vaccines and specific antiviral treatments are not available, vector prevention and control strategies have been crucial in limiting the rise in dengue cases and the severity of dengue epidemics [3]. Therefore, it is essential to control the populations of DENV vector mosquitoes, particularly Aedes aegypti and Aedes albopictus, and limit their spread to new areas to prevent the transmission of DENV [3].

In recent years, Nepal has witnessed an increasing incidence of dengue fever cases. Almost every monsoon season, dengue outbreaks have been documented in Nepal, predominantly in the Terai region. Major outbreak years include 2007, 2009, 2010, 2013, 2016, 2019, 2020 and 2022 [4, 5]. Local epidemiological studies underscore the variability in outbreak characteristics, including serotype predominance and healthcare impacts. The serotypes have shifted from dengue virus serotype − 1 (DENV-1) in 2010, DENV-2 in 2013, DENV-1 in 2016, DENV-2 in 2019 and DENV-1, DENV-2 and DENV-3 in 2022 outbreak [6, 7]. Variations in the duration, serotype distributions, and effects on the healthcare system have been observed in these outbreaks [8, 9]. The fact that Dengue Fever keeps recurring annually and the increasing number of cases during each outbreak implies that the current vector control measures are likely inadequate and should be enhanced [9–11].

To address the recurrent nature of dengue outbreaks, it is essential to consider both intrinsic and extrinsic factors which signify the combination of an environmental, biological and socio- economic factors. Research indicates that inadequate vector control measures and environmental factors, such as climate change, increased unplanned urbanization leading to poor sanitation and waste management, and inadequate water storage, contribute to persistence dengue transmission [12–15]. The circulation of different dengue virus serotypes, combined with increasing global mobility, further complicates the control of the disease. Human factors, such as migration and travel introduce the virus to new areas, fueling outbreaks in regions previously unaffected [16–18]. Socioeconomic disparities, including limited access to healthcare and inadequate vector control measures, also contribute to recurrent outbreaks in low-income areas [19, 20].

The effective management and prevention of dengue fever depends not only on the efforts of healthcare professionals and policymakers but also on the knowledge, attitudes and practices (KAP) of the community members who are at risk [21, 22]. A lack of proper understanding of dengue transmission and necessary preventive measures can increase the risk of spreading dengue fever [23]. Evidence suggests that community education may be more effective than solely relying on insecticide spraying to decrease mosquito breeding grounds [24, 25]. Communities with greater awareness of dengue transmission and prevention are more likely to adopt practices like removing stagnant water, using mosquito nets, and seeking timely medical care [26]. Positive attitudes toward public health interventions, such as indoor spraying and community clean-up campaigns, enhance the success of outbreak management efforts [27, 28]. Behavioral change driven by effective communication and education, is essential for reducing the disease burden [29]. Ultimately, controlling dengue requires not only improved public health infrastructure but also an informed and engaged community willing to take preventive action [25, 30].

Thus, to develop lasting public health interventions for dengue that affect individuals with diverse socio-economic and cultural backgrounds across Nepal’s different altitudinal regions, it is critical to identify and comprehend the knowledge, attitude, and practices (KAP) of the population towards dengue virus and its vectors [23, 31]. Despite the increasing incidence of dengue fever each year, very few research studies have been conducted to assess the knowledge, attitude and practice of dengue fever among community people globally and in Nepal. In this context, this study aimed to assess the knowledge, attitudes and practices of dengue fever among community people of one of the core endemic cities (i.e., Lalitpur Metropolitan City) of Nepal. Lalitpur is one of the three major cities in the valley and is the administrative center of Lalitpur District. The Lalitpur metropolitan city is an urban city of a total of 29 wards, consisting of 77,159 households and 284922 total population [32]. The city is centrally located and surrounded by all urban municipalities. The city consists of numerous ponds and stone water spouts ( a traditional water system where water flows from stone–carved spouts) [33]. The city is characterized by rapid urbanization, a dense population, and mixed land-use patterns, all of which contribute to increased public health challenges, including vector-borne diseases like dengue fever [34–36].

## Methods

### Study design and site description

The study employed a descriptive cross-sectional study design [37] using a concurrent mixed method approach [38] to assess the knowledge, attitudes and practices of dengue fever among community people of Lalitpur Metropolitan City. After Kathmandu and Pokhara, Lalitpur is the third-largest city in Nepal with 29 wards, 77159 households and a population of 284922 [34]. It is situated near the south side of the Bagmati River and the south-central section of the Kathmandu Valley [33]. Lalitpur has become one of South Asia’s fastest-developing cities as a result of its rapid urbanization and population growth [35]. Lalitpur was one of the districts that had a high number of dengue infections noted in Bagmati Province during the 2022 outbreak [36]. Thus, the Lalitpur Metropolitan City was selected purposively as the study site. Three randomly selected wards of the Lalitpur Metropolitan City were visited to collect the data for the study. The community people (above 18 years of age) residing in the Lalitpur Metropolitan City were the study population.

### Sample size and sampling method

The sample size was calculated, assuming the 50% population having knowledge which is a standard approach to be used where the true prevalence is unknown. This approach provides the maximum variability and ensures an adequate sample size to ensure an accurate prediction [39].

Accordingly, the sample size was estimated with the following parameters, using the cross-sectional study formula [40], and the calculated sample size was 634.

Prevalence rate (p) = 50%

q = 50%

Type I error (α) = 5%

Z1-α/2 = 1.959964

Sample size (n) = 384

Non-response rate = 10%

Participation rate = 90%

Design effect = 1.50

The sample size was calculated using the formula: [40]

- Minimum Sample Size (N) = z^2^pq/e^2^ * (1 + Non-response rate) = 423
- Final Sample size (N_final_) = z^2^pq/e^2^ * (1 + Non-response rate) * Design effect = 634

The total sample size taken for the study was 636, with 212 samples in each ward.

The household head was the sampling unit. Multistage cluster sampling techniques were applied. Lalitpur Metropolitan City was initially selected through purposive sampling. Out of its 29 wards, 10% which accounted for 3 wards that were chosen, considering the feasibility and available resources. Given the scope of the study and the available resources, this sample size was deemed adequate to capture a representative range of experiences while allowing for comprehensive data collection. Hence, a total of 3 wards from the Lalitpur Metropolitan City were selected through simple random technique to minimize bias and ensure that the sample was reflective of the larger population. After selecting the wards, the clusters were divided so that each cluster had around 215 households. Then, one cluster from each ward was selected through a simple random sampling (lottery method) to capture the diversity within wards. This method was chosen to reduce selection bias and to capture variability within the ward. The entire households from the selected cluster were visited to collect the data. A member of the household who was willing to provide information was purposively chosen and interviewed for data collection.

For the qualitative study, twenty in-depth interviews were conducted among the local stakeholders i.e., local leaders and Female Community Health Volunteers (FCHVs). The participants for the in-depth interview were selected through a purposive judgmental sampling technique [41]. The selection criteria focused on individuals directly involved in implementing dengue prevention and control initiatives. Local leaders and Female Community Health Volunteers (FCHVs) were actively involved in implementing public health interventions at the community level, such as dengue awareness and search-and-destroy programs. Therefore, both groups were identified as participants possessing specific knowledge relevant to the study objectives. The number of participants for the qualitative interviews was determined through the information saturation level [42].

### Study variables

Knowledge about dengue, attitude towards dengue and preventive and control practices against dengue fever are the dependent variables. The independent variables in the study are age, gender, marital status, ethnicity, religion, education, occupation, health-related professionals (either an academic health background or actively working in health-related roles), types of family, residency status, previous experience of dengue fever and socio- economic status.

The ethnicity includes Brahmin/Chhetri (historically privileged in the caste system), Janajati (an indigenous group with distinct culture), Madhesi (people from the Terai region with cultural ties to northern India), Muslim (people following Islamic traditions) and Dalit (historically marginalized groups facing social and economic challenges).

Regarding education status, “literate” refers to individuals who can read and write but have not received formal schooling. “Illiterate” denotes those who are unable to read or write. In contrast, “all other educational levels” signify individuals who have undergone formal education, which includes primary, secondary, or higher education within an established schooling system.

The socio-economic status of the household was determined by the Modified Kuppuswamy scale [43]. This scale uses three aspects: education of household head, occupation of the household health and monthly family income. Based on the total score obtained, socio-economic class is categorised as upper, middle and lower class.

### Data collection

The data was collected from April 2023 to June 2023. The structured questionnaire was used as a data collection tool for quantitative study. The data collection tools were prepared based on the literature review. The questionnaire was divided into two sections. Section A sought information about the socio-demographic information of the participants, and Section B asked questions related to knowledge, attitude, and practice. The questions related to knowledge, attitude, and practice were adopted from a validated tool of a previous study conducted in Nepal [44]. The Cronbach’s alpha coefficients of the KAP domains obtained from the study were 0.8, 0.7, and 0.8, respectively [44].

Firstly, the enumerators identified the study participants with whom the interview would be conducted. Following identification, the procedure of providing information and gaining consent was carried out. Data were collected using structured questionnaires, and face-to-face interview approaches were used for data collection after obtaining verbal and written consent. In the same way, twenty in-depth interviews were conducted using a semi-structured interview guideline. The information from the participants through face-to-face interviews was captured using a password-protected audio recording device, and field notes were also taken.

The data collection process was performed by trained enumerators who held a Bachelor’s degree in Public Health and had prior experience in data collection. They received training on the study’s objectives and data collection methods.

### Data management and analysis

All completed questionnaire forms were manually edited and coded before being entered into the Excel. After cleaning the data in the Excel, data analysis was done using the STATA 13 2022 version. For the weighted analysis, the normalized weight was calculated. Since all clusters had equal sample sizes, the normalized weight for each cluster was 1. As a result, there was no difference between the weighted and unweighted analysis, so only the weighted analysis was reported throughout the study. The overall KAP score of the participants regarding DF was determined by assigning a score of one for each correct answer and a score of zero for each incorrect answer. In addition, “Do not know” (DNK) responses were treated as incorrect and also given a score of zero. These individual scores were then added together based on the number of questions in the questionnaire to obtain a potential total score. The Knowledge, attitudes and practices variables were dichotomized based on an 80% cutoff point [44–47].

Following the completion of the descriptive analysis, we conducted a bivariate analysis to measure the relationship between the dependent variable and each independent variable individually. For every independent variable, we computed unadjusted odds ratios (OR) along with their corresponding 95% confidence intervals (CI). Subsequently, we assessed the p-values obtained from this initial bivariate analysis, and we considered variables with *p*-values below 0.25 for inclusion in the multivariable model. After deciding based on the p-values, we conducted a multicollinearity test using the variance inflation factor (VIF) for all eligible variables in the multivariate logistic regression [48, 49]. Only variables with a VIF less than two were included in the regression analysis [50]. Notably, all the variables in our study exhibited VIF values below two. Therefore, all eligible variables were incorporated into the final multivariable logistic regression model. We set the significance level at 5%. And for the qualitative data, the data collected from the interviews were translated. The translated documents were used for the analysis of the data. The thematic analysis was done on qualitative data by utilizing the Qualitative Data Analysis in Dedoose software. Following this, the triangulation of the results obtained from both qualitative and quantitative data was done to increase the validity of the findings [37].

## Result

### Socio-demographic characteristics of the study participants

The mean age of the participants was 36.58 years, with a standard deviation of 13.24 years. The gender distribution showed that 51.42% of the respondents were male. Ethnicity revealed that the largest ethnic group among the respondents was “Janajati,” accounting for 50.47% of the total. In terms of religion, the majority of respondents identified as “Hindu” (74.06%), followed by “Buddhist” (18.24%). Education-wise, the highest proportion (39.78%) had a “Secondary” level of education, followed by “Higher secondary” (32.70%). Regarding marital status, most respondents (65.72%) reported being “Married,” while 31.60% were “Unmarried. In terms of health-related matters, 2.20% of the respondents reported being “persons related to health,” while the majority (97.80%) answered “No.” The distribution of family types showed a near-equal split between “Nuclear” (50.31%) and “Joint/ Extended” (49.69%) families. In terms of occupation, the largest group of respondents was engaged in “Business” (38.78%), followed by “Service” (32.65%). Socio-economic status indicated that the majority of respondents (86.64%) fell into the “Middle” category, while smaller percentages were classified as “Low” or “High”. Lastly, regarding residency, the majority of respondents (54.72%) owned their homes, while 45.28% resided in rented accommodations (Table 1).

**Table 1:** Socio-demographic characteristics of the study participants

Variable	Characteristics	Frequency (*N* = 636)	Percentage
**Age**	18–29	220	34.6
	30–44	246	38.7
	45–60	139	21.8
	> 60	31	4.9
**Gender**	Male	327	51.42
	Female	309	48.56
**Ethnicity**	Dalit	15	2.36
	Janajati	321	50.47
	Madhesi	55	8.65
	Muslim	7	1.10
	Brahmin/Chhetri	230	36.16
	Others	8	1.26
**Religion**	Buddhist	116	18.24
	Christian	30	4.72
	Hindu	471	74.06
	Muslim	11	1.73
	Other	8	1.26
**Education**	Illiterate	7	1.10
	Literate	36	6.76
	Primary	48	14.31
	Secondary	162	39.78
	Higher Secondary	208	32.70
	Above higher secondary	175	27.52
**Marital Status**	Unmarried	201	31.60
	Married	418	65.72
	Widow	17	2.67
**Person related to health**	Yes	14	2.20
	No	622	97.80
**Types of Family**	Nuclear	320	50.31
	Joint/ Extended	316	49.69
**Occupation**	Student	15	2.35
	Self-employed	22	3.45
	Business	247	38.78
	Service	208	32.65
	Retired	7	1.10
	Unemployed	22	3.45
	Homemaker	85	13.34
	Other	31	4.87
**Socio Economic Status**	Low	59	9.28
	Middle	551	86.64
	High	26	4.09
**Residency**	Own home	348	54.72
	Rent	288	45.28

### Report of dengue fever within the past two years in the community

Among the 636 households studied, 34.12% (*n* = 217) reported suffering from dengue infections in the past two years, while 29.87% (*n* = 190) of respondents had personally been infected. The qualitative findings echoed this high prevalence, with participants explaining that dengue was widespread in the previous year, leading to numerous infections. One respondent shared,

*“Yes*,* my whole family had dengue. Symptoms varied from person to person.” (IDI 5)*

### Knowledge, attitude, and practice regarding dengue fever

In terms of knowledge, 64.94% (*n* = 413/636) of the respondents reported having high knowledge about dengue fever and, a large majority i.e., 91.51% (*n* = 582/636) expressed a positive attitude towards dengue fever, indicating a favorable perception and awareness of its importance. The participants in the qualitative interviews, local leaders and volunteers were responsible for making the people aware of dengue fever and they were well aware of dengue fever. One participant explained that;

*"We make people aware by saying*,* dengue is a communicable disease. Its symptoms include high fever and fatigue. Though it looks like a mild illness*,* it can even cause death. For treatment*,* we can take paracetamol and a wet cloth remedy to cool the fever. It is transmitted from mosquitoes which are most active two hours before sunrise and sunset. We should be cautious and avoid going into the field to minimize exposure to mosquitoes."   (IDI 2)*

Although the majority of respondents in the community reported having high knowledge of dengue fever and expressed a positive attitude toward prevention, only 49.84% (*n* = 317/636) were involved in preventive practices. This gap between knowledge and action was also reflected in the qualitative interviews, where participants expressed a lack of adherence to preventive measures in their communities. One participant in the interview explained,

*“The people in the area are aware. People don’t follow preventive measures even though they are well aware of the control and preventive procedures. Thus*,* we are doing the awareness initiative in this ward.”  (IDI 19)*

Among the total of 217 respondents who reported infected with dengue, 141 individuals (64.97%) reported that they visited a medical doctor for disease diagnosis. Regarding the treatment of dengue, out of the 217 respondents, 123 individuals (56.68%) stated that they purchased medicines from a pharmacy without a prescription from a medical doctor. Qualitative findings also revealed personal experiences with dengue. One participant shared,

*“Me and my son got dengue. I got dengue during Teej (a traditional Hindu festival celebrated primarily by women) and had a fever. When I went to the hospital for a checkup*,* they diagnosed me as dengue.”  (IDI 13)*

### Source of information about dengue

According to the data, television (90.57%) and social media (83.49%) were reported as being the most frequently used sources of information, with 576 and 531 respondents respectively reported primarily relying on these platforms. The radio (67.55%) and neighbors (69.81%) were also significant sources of information, with 429 and 444 individuals respectively mentioning them. Health workers (42.6%) and miking (37.74%) were reported by 271 and 240 respondents respectively. Teachers (14.31%) and children (4.87%) were mentioned by 91 and 31 individuals respectively. These findings suggest that while traditional media like television and radio still play a significant role, social media has become an increasingly popular source of information for a large portion of the respondents (Table 2). The qualitative interviews supported these insights, revealing that information about dengue comes from a variety of sources, including local community networks, media outlets, personal experiences, and formal awareness programs. This showed the role of both formal and informal sources in raising awareness about dengue.

One participant stated,

*“At first*,* I heard about it from the news*,* and after being involved in an awareness program*,* I learned many things. It’s only been 2 or 3 years since I first heard about it.” (IDI 9)*

**Table 2:** Source of information about dengue

Source	Frequency	Percent
Radio	429	67.55
TV	576	90.57
Health Worker	271	42.6
Miking	240	37.74
Neighbors	444	69.81
Teacher	91	14.31
Children	31	4.87
Social Media	531	83.49
Others	18	3.49

multiresponse

### Factors associated with the knowledge, attitude and practice

#### Association of knowledge on dengue fever with socio-demographic characteristics

In the bivariate analysis, several variables such as age, gender, ethnicity, education, socio-economic status, residency, and personal experience of getting dengue fever demonstrated statistical significance with having a high knowledge of dengue fever. However, when considering all of these variables together in the multivariable analysis, only gender, residency, and personal experience of contracting dengue fever remained significantly associated with knowledge about dengue fever. The lack of significance for variables like age, ethnicity, education and socioeconomic status may be due to other contextual factors specific to the study population.

According to multivariable analysis, in comparison to males, females had 1.61 times more knowledge of dengue fever (AOR: 1.61, 95% CI (1.12–2.31)). Likewise, in comparison to those who own homes, individuals who are at rent were 48% (AOR: 0.52, 95% CI (0.36–0.77)) less likely to possess the knowledge being studied. Furthermore, compared to those who have not experienced dengue, individuals with personal experience were 2.16 times (AOR: 2.16, 95% CI (1.44–3.26)) more likely to possess the knowledge being studied (Table 3).

**Table 3:** Factors associated with knowledge of dengue fever among community people

Variable	Knowledge (*N* = 413)	Unadjusted		Adjusted	
		OR (95% CI)	*P* value	OR (95% CI)	*P* value
**Age**		0.99 (0.97–1.00)	0.199*	0.99 (0.97–1.01)	0.497
**Gender**					
Male	194 (46.97)	Ref	0.002**	Ref	0.009**
Female	219 (53.03)	1.66 (1.199- 2.320)		1.61 (1.12–2.31)	
**Ethnicity**					
Dalit	8 (1.94)	Ref	0.075*	Ref	
Janajati	200 (48.43)	1.44 (0.511–4.08)		0.79 (0.26–2.39)	0.68
Madhesi	36 (8.72)	1.65 (0.52–5.27)		1.28 (0.38–4.33)	0.68
Muslim	2 (0.48)	0.35 (0.05–2.40)		0.36(0.46–2.82)	0.33
Brahmin/ Chhetri	160 (38.74)	2 (0.69–5.72)		1.07 (0.35–3.30)	0.89
Other	7 (1.69)	6.125 (0.59–62.82)		3.92 (0.35–43.7)	0.26
**Religion**					
Buddhist	71 (17.19)	Ref	0.49		
Christian	18 (4.36)	0.95 (0.41–2.15)			
Hindu	314 (76.03)	1.26 (0.83–1.92)			
Muslim	5 (1.21)	0.52 (0.15–1.83)			
Others	5 (1.21)	1.05 (0.24–4.63)			
**Education**					
Illiterate	4 (0.97)	Ref	0.0026**		
Literate (Informal education)	19 (4.60)	0.83 (1.63–4.29)		1.49 (0.25–8.59)	0.65
Primary	24 (5.81)	0.75 (0.15–3.71)		1.39 (0.25–7.73)	0.70
Secondary	96 (23.24)	1.09 (0.23–5.03)		1.94 (0.37–10.18)	0.43
Higher Education	138 (33.41)	1.47 (0.32–6.78)		2.44 (0.45–13.22)	0.29
Higher study graduates	132 (31.96)	2.30 (0.49–10.69)		3.91 (0.72–21.20)	0.11
**Marital Status**					
Unmarried	133 (32.20)	Ref	0.557		
Married	271 (65.62)	0.94 (0.66–1.34)			
Widow/ Divorced/ Separated	9 (2.18)	0.57 (0.21–1.55)			
**Health-related professional**					
Yes	10 (2.42)	Ref	0.608		
No	403 (97.58)	0.73 (0.22–2.37)			
**Types of Family**					
Nuclear	204 (49.39)	Ref	0.528		
Joint/ extended	209 (50.61)	1.11 (0.80–1.53)			
**Socio Economic Status**					
Low	31 (7.51)	Ref	0.029**	Ref	
Middle	361 (87.41)	1.71(0.99–2.94)		1.72 (0.95–3.10)	0.071
High	21 (5.08)	3.79(1.26–11.40)		2.69 (0.84–8.61)	0.095
**Residency**					
Own home	246 (59.56)	Ref	0.001**	Ref	
Rent	167 (40.44)	0.57 (0.41–0.794)		0.52 (0.36–0.77)	0.001**
**Having personal experience of dengue**					
No	267(59.87)	Ref	0.000**	Ref	
Yes	146 (76.84)	2.22 (1.51–3.27)		2.16 (1.44–3.26)	0.000**

*P value < 0.25

**P value < 0.05

The qualitative interview also supported the findings generated from this analysis. One of the research participants shared her experiences as.

“*In previous years*,* when we informed the community about dengue*,* they used to ignore it and were unaware. However*,* after experiencing a dengue epidemic*,* people became more alert to the disease……………. Nowadays*,* there is greater awareness among people*,* who now understand that dengue is a significant and more severe threat compared to COVID-19.*”   (*IDI 4*)

#### Association of attitude on dengue fever with socio-demographic characteristics

In the initial bivariate analysis examining the relationship between attitude and socio-demographic variables, age and residency were found to be statistically significant. However, when these variables were collectively assessed in the multivariable analysis, none exhibited a significant association with having a positive attitude towards dengue fever (Table 4).

**Table 4:** Factors associated with attitude on dengue among community people

Variable	Attitude (*N* = 582)	Unadjusted		Adjusted	
		OR (95% CI)	*P* value	OR (95% CI)	*P* value
**Age**		1.02 (1.002–0.05)	0.022**	1.02 (0.99–1.04)	0.063
**Gender**					
Male	299 (91.44)	Ref	0.946		
Female	283 (91.59)	1.01 (0.58–1.78)			
**Ethnicity**					
Dalit	13 (86.67)	Ref	0.329		
Janajati	294 (91.59)	1.67 (0.35–7.81)			
Madhesi	48 (87.27)	1.05 (0.19–5.69)			
Muslim	5 (71.43)	0.38 (0.04–3.52)			
Brahmin/ Chhetri	214 (93.04)	2.05 (0.42–9.92)			
Other	8 (100.00)	1			
**Religion**					
Buddhist	104 (89.66)	Ref	0.60		
Christian	27 (90.00)	1.03 (0.27–3.94)			
Hindu	434 (92.14)	1.35 (0.68–2.68)			
Muslim	9 (81.82)	0.51 (0.10–2.68)			
Others	8 (100.00)	1			
**Education**					
Illiterate	6 ( 85.71)	Ref	0.27		
Literate (Informal education)	35 (97.22)	5.8 (0.31-106.43)			
Primary	40 (83.33)	0.83 (0.87–7.89)			
Secondary	151 (93.21)	2.28 ( 0.25–20.72)			
Higher Education	191 ( 91.83)	1.87 (0.21–16.44)			
Higher study graduates	159 (90.86)	1.65 (0.18–14.63)			
**Marital Status**					
Unmarried	181 (90.05)	Ref	0.64		
Married	385 (92.11)	1.28 (0.71–2.30)			
Widow/ Divorced/ Separated	16 (94.12)	1.76 (0.22–14.04)			
**Health-related Professionals**					
Yes	13 (92.86)	Ref	0.85		
No	569 (91.48)	0.82 (0.10–6.43)			
**Types of Family**					
Nuclear	294 (91.88)	Ref	0.73		
Joint/ extended	288 (91.14)	0.90 (0.52–1.58)			
**Socio Economic Status**					
Low	53 (89.83)	Ref	0.71		
Middle	503 (91.29)	1.18 (0.48–2.90)			
High	26 (100.00)	1			
**Residency**					
Own home	324 (93.10)	Ref	0.115*	Ref	
Rent	258 (89.58)	0.63 (0.36–1.11)		0.74(0.41–1.33)	0.321
**Having Personal experience of dengue**					
No	406 (91.03)	Ref	0.508		
Yes	176 (92.63)	1.23(0.65–2.33)			

*P value < 0.25

**P value < 0.05

#### Association of practice on dengue fever with socio-demographic characteristics

In the initial bivariate analysis examining the relationship between preventive practice and socio-demographic variables, gender, ethnicity, education, socio-economic status, residency, knowledge and attitude were found to be statistically significant. While considering all of these variables together in the multivariable analysis, only gender, residency and attitude remained significantly associated with preventive practice on dengue fever.

After including variables that showed significant association in the final regression model, males (AOR: 0.67, 95% CI (0.48–0.93), people living on rent (AOR: 1.42, 95% CI (1.01–2.02), and having a positive attitude on dengue (AOR: 1.83, 95% CI (1.00-3.34) are more likely to follow the preventive practice in comparison to their counterparts (Table 5).

**Table 5:** Factors associated with practice of dengue among community people

Variable	Practice (*N* = 317)	Unadjusted		Adjusted	
		OR (95% CI)	*P* value	OR (95% CI)	*P* value
**Age**		1.00 (0.99–1.01)	0.30		
**Gender**					
Male	179 (54.74)	Ref	0.011**	Ref	
Female	138 (44.66)	0.66 (0.48–0.91)		0.67 (0.48–0.93)	0.01**
**Ethnicity**					
Dalit	7 (46.67)	Ref	0.0007**	Ref	
Janajati	159 (49.53)	1.12 (0.39–3.16)		1.16 ( 0.39–3.48)	0.78
Madhesi	15 (27.27)	0.42 (0.13–1.38)		0.41 (0.11–1.35)	0.15
Muslim	4 (57.14)	1.5 (0.24–9.29)		1.21 (0.18–7.52)	0.84
Brahmin/ Chhetri	129 (56.09)	1.45 (0.51–4.15)		1.49 (0.49–4.48)	0.48
Other	3 (37.50)	0.68 (0.12–3.96)		0.63 (0.09–3.63)	0.61
**Religion**					
Buddhist	51 ( 43.97)	Ref	0.29		
Christian	15 (50.00)	1.27 (0.57–2.84)			
Hindu	243 (51.59)	1.35 (0.92–2.04)			
Muslim	5 (45.45)	1.06 (0.30–3.67)			
Others	3 (37.50)	0.76 (0.17–3.35)			
**Education**					
Illiterate	4 (57.14)	Ref	0.032**	Ref	
Literate (Informal education)	13 (36.11)	0.42 (0.08–2.19)		0.25 (0.04–1.46)	0.12
Primary	19 (39.58)	0.49 (0.09–2.44)		0.29 (0.05–1.61)	0.15
Secondary	78 (48.15)	0.69 (0.15–3.21)		0.37 (0.07–1.97)	0.24
Higher Education	109 (52.40)	0.82 (0.18–3.78)		0.43(0.08–2.24)	0.32
Higher study graduates	94 (53.71)	0.87 (0.18-4.00)		0.46 (0.08–2.41)	0.36
**Marital Status**					
Unmarried	95 (47.26)	Ref	0.63		
Married	214 (51.20)	1.17 (0.83–1.63)			
Widow/ Divorced/ Separated	8 (47.06)	0.99 (0.36–2.67)			
**Health-related Professionals**					
Yes	9 (64.29)	Ref	0.28		
No	308 (49.52)	0.54 (0.18–1.64)			
**Socio Economic Status**					
Low	21 (35.59)	Ref	0.0443**	Ref	
Middle	285 (51.72)	1.9 (1.10–3.38)		1.76 (0.98–3.17)	0.05
High	11 (42.31)	1.3 (0.51–3.40)		1.29 (0.48–3.48)	0.60
**Residency**					
Own home	161 (46.26)	Ref	0.047**	Ref	
Rent	156 (54.17)	1.37 (1.00-1.87)		1.42 (1.01–2.02)	0.04**
**High Knowledge**					
No	121 (54.26)	Ref	0.102*	Ref	
Yes	196 (47.46)	0.76 (0.54–1.05)		0.72 (0.50–1.03)	0.07
**Positive Attitude**					
No	21(38.89)	Ref	0.095*	Ref	
Yes	296 (50.86)	1.62 (0.91–2.87)		1.83 (1.00-3.34)	0.04**

*P value < 0.25

**P value < 0.05

The study revealed a contrast between quantitative and qualitative findings regarding the knowledge and practices of dengue prevention among people living in rented houses compared to those living in their own homes. Quantitative data indicated that, although knowledge levels were lower among those in rented housing, they exhibited more positive behaviors related to dengue prevention than homeowners. However, qualitative interviews provided a different perspective. Most participants observed that individuals living in rented homes had low knowledge, which contributed to poor preventive practices.

One key informant explained,

*“….the people in the rent tend to collect the rainwater in the open container*,* larvae are being seen in those containers. When we go to destroy the larva from the container*,* they won’t let to do so as the water was scarce.” (IDI16)*

## Discussion

This study provided a comprehensive analysis of the occurrence of dengue, as well as the knowledge, attitudes, and practices (KAP) related to dengue, in Lalitpur metropolitan city which witnessed a notable dengue outbreak in 2022. Furthermore, the study examined the experiences and measures implemented during the dengue epidemic.

The findings of this study indicated that 34% of the households studied had experienced dengue infections, while 29% of the respondents reported having been infected with dengue within the two years before the study. This is not unexpected considering that Lalitpur Metropolitan City, being an urban area in Nepal, is at a heightened risk for a higher prevalence of dengue cases. This conclusion finds further support in the dengue situational report for the year 2022, which documented a substantial number of dengue cases in the Lalitpur district [5]. It’s noteworthy that a hospital-based study conducted in 2019 reported a prevalence rate of 63.09%. The disparities in these findings may be attributed to the fact that the 2019 study employed rapid diagnostic kits (RDTs) to identify DENV infections in a tertiary healthcare setting [51].

In the current study, it was found that 64% of the participants exhibited a good knowledge of Dengue fever, a proportion that surpasses the results of similar studies previously conducted in Nepal [31, 44, 52, 53]. However, it is important to note that a hospital-based study conducted in Nepal showed a higher knowledge of dengue fever [54]. Comparatively, the studies conducted in other countries such as India, Malaysia, Indonesia and Jamaica have shown lower levels of knowledge regarding dengue [22, 55–57]. The higher knowledge in this study may be attributed to the fact that Lalitpur, being an urban center, benefits from greater access to health information through various media outlets, which can increase awareness.

Regarding attitudes, a significant majority of the participants in this study displayed a highly positive attitude (91.51%). This outcome aligns with another study conducted in Nepal, which also reported high levels of positive attitude [31, 44]. However, the contrast findings were observed in the study among the police personnel of Nepal which reported only 46% [53]. The variations in attitudes between different studies could be influenced by differences in population characteristics, such as education, profession, and exposure to health education campaigns. In this context, it’s possible that the population in this study, living in an urban setting, may have had more opportunities for health education compared to other groups, such as police personnel.

However, despite the high levels of knowledge and positive attitude, the current study revealed that only approximately 50% of the participants reported adopting positive behaviors. This gap between knowledge, attitude and practices is concerning and in contrast to the other studies conducted in Nepal and, Sri Lanka which found that participants exhibited good practices related to control measures, despite having a lower level of knowledge about Dengue fever [44, 53, 58]. Likewise, some studies conducted in India demonstrated the discrepancy that the majority reported good knowledge of dengue and poor preventive practices [59]. Some studies in Pakistan, Lao PDR and India have shown good knowledge about dengue related to good preventive practices [60–62]. The variations in knowledge, attitudes and practice observed between different studies can be attributed to a combination of factors, including the specific study population, cultural and contextual differences, the timing of the studies, and the methods used for data collection and assessment. These factors highlight the complex interplay of individual, societal, and environmental factors in shaping health-related behaviors.

Television and social media were identified as the primary sources of dengue-related information, consistent with previous studies in Nepal [31, 44] and other countries like Jamaica, India, and Malaysia [56, 60, 63]. Our finding consistent with these findings emphasizes the importance of these platforms in health communication efforts, especially in urban areas where access to technology is more widespread.

Regarding healthcare-seeking behavior, just under two-thirds (64.97%) of households preferred consulting medical doctors for diagnosis and treatment. Furthermore, over 50% of respondents mentioned that they or their families purchased medications directly from local pharmacies without obtaining a prescription from healthcare professionals. This behavior is highly worrisome, as it has the potential to lead to severe and life-threatening complications [52]. This findings aligns with a study conducted in Myanmar, where 46.1% of individuals expressed a preference for self-medication as their initial approach [64]. These findings highlight the importance of promoting safe and responsible healthcare practices to minimize the risks associated with self-medication.

The results revealed a significant association between gender, residency status, and prior dengue experience with the participant’s level of understanding regarding dengue fever. Comparable findings were observed in a previous study conducted in Nepal, where education level and residential area exhibited statistical significance concerning dengue knowledge [44]. Conversely, another study within Nepal highlighted that ethnicity and family type were significantly linked to knowledge about dengue fever [52]. Furthermore, a different study in Haryana India indicated that caste and socioeconomic status had significant associations with knowledge [65]. On the other hand, research conducted in other country such as in India and Malaysia found no significant links between knowledge and socio-demographic characteristics [60, 63]. The observed differences between studies may be due to variations in the study populations and the timing of data collection. For instance, socio-cultural norms and local perceptions of disease risk may differ across regions and ethnic groups, affecting knowledge levels.

In the current study, no statistically significant association was observed between attitude and socio-demographic characteristics. This finding is in line with a similar study conducted in Nepal [44] and Indonesia [55]. However, the study in Malaysia showed attitude was significantly associated with education level and occupation [63]. These differences may be attributed to contextual factors, such as the role of education in shaping health-related attitudes in different cultural settings.

Regarding the relationship between preventive practices and independent variables, factors such as gender, residency, and attitude were found to be significantly linked to dengue fever preventive practices in this study. However, different results were reported in another study conducted in Nepal [44, 52], where no significant association was observed between having knowledge and adhering to preventive measures. This contradicts findings from another study, where individuals with high knowledge reported following good preventive practices [63, 66]. The finding showing a significant association between attitude and practice is in line with a study conducted in Indonesia [55], highlighting the importance of fostering positive attitudes to drive behaviour change.

While this study has notable strengths, it also has certain limitations. A key limitation is that being a cross-sectional study, it captures relationships at a single point in time. Additionally, the sampling method as selecting one cluster per ward and one individual per household may restrict the representativeness and generalizability of the findings. The results are specific to urban environments, making it unsuitable to generalize them to rural areas or the national level. Furthermore, the study did not assess the actual implementation of dengue preventive measures, meaning the conclusions are based solely on participants’ responses, which may be subject to social desirability bias. Despite these limitations, the study provides valuable insights into community knowledge, attitudes, and practices regarding dengue fever, which are essential for effective prevention and control of dengue outbreaks.

## Conclusion

Despite community members demonstrating adequate knowledge and positive attitudes, the study found a gap between awareness and the actual implementation of preventive measures for dengue management. While participants were well-informed about dengue, they did not consistently adopt effective preventive practices. However, a positive attitude was shown to encourage adherence to these measures. This highlights the need for strategies that bridge the gap between knowledge and practice. Strengthening social mobilization and communication efforts through Information, Education, and Communication (IEC) and Behavior Change Communication (BCC) programs is essential for improving dengue prevention behaviors.

## Data Availability

The datasets used and/or analyzed during the current study are available from the corresponding author upon reasonable request.

## References

[1] Stanaway JD, Shepard DS, Undurraga EA, Halasa YA, Coffeng LE, Brady OJ, et al. The global burden of dengue: an analysis from the Global Burden of Disease Study 2013. Lancet Infect Dis. 2016;16(6):712–23. 26874619 10.1016/S1473-3099(16)00026-8 PMC5012511

[2] Halstead SB. Dengue vaccine development: a 75 solution? Lancet. 2012;380(9853):1535–6. 22975339 10.1016/S0140-6736(12)61510-4

[3] WHO. Comprehensive guidelines for prevention and control of dengue and dengue haemorrhagic fever. New Delhi: World Health Organization, Regional Office for South-East Asia; 2011.

[4] Khetan RP, Stein DA, Chaudhary SK, Rauniyar R, Upadhyay BP, Gupta UP, et al. Profile of the 2016 dengue outbreak in Nepal. BMC Res Notes. 2018;11(1):1–6. 29970132 10.1186/s13104-018-3514-3 PMC6029055

[5] Epidemiology and Disease Control Division (EDCD). Situation update of dengue 2022 (As of 31th Dec,2022). 2022.

[6] Poudyal P, Sharma K, Dumre SP, Bastola A, Chalise BS, Shrestha B, et al. Molecular study of 2019 dengue fever outbreaks in Nepal. Trans R Soc Trop Med Hyg. 2021;115(6):619–26. 32987406 10.1093/trstmh/traa096

[7] Gupta BP, Lamsal M, Chaulagain S, Rauniyar R, Malla R, Shrestha S, et al. Emergence of dengue in Nepal. VirusDis. 2018;29(2):129–33. 10.1007/s13337-018-0439-3 PMC6003061 29911144

[8] Malla S, Thakur GD, Shrestha SK, Banjeree MK, Thapa LB, Gongal G, et al. Identification of all Dengue Serotypes in Nepal. Emerg Infect Dis. 2008;14(10):1669–70. 18826846 10.3201/eid1410.080432 PMC2609890

[9] Rimal S, Shrestha S, Pandey K, Nguyen TV, Bhandari P, Shah Y, et al. Co-circulation of dengue virus serotypes 1, 2, and 3 during the 2022 dengue outbreak in Nepal: a cross-sectional study. Viruses. 2023;15(2):507. 36851721 10.3390/v15020507 PMC9958792

[10] Gunasekara T, Velathanthiri V, Weerasekara M, Fernando S, Peelawattage M, Guruge D, et al. Knowledge, attitudes and practices regarding dengue fever in a suburban community in Sri Lanka. Galle Med J. 2012;17(1):10.

[11] Pandey BD, Pandey K, Dumre SP, Morita K, Costello A. Struggling with a new dengue epidemic in Nepal. Lancet Infect Dis. 2023;23(1):16–7. 36442489 10.1016/S1473-3099(22)00798-8

[12] Bhatt S, Gething PW, Brady OJ, Messina JP, Farlow AW, Moyes CL, et al. The global distribution and burden of dengue. Nature. 2013;496(7446):504–7. 23563266 10.1038/nature12060 PMC3651993

[13] Morin CW, Sellers S, Ebi KL. Seasonal variations in dengue virus transmission suitability in the Americas. Environ Res Lett. 2022;17(6):064042.

[14] Mohapatra S, Aslami A. Knowledge, attitude and practice regarding dengue fever among general patients of a rural tertiary-care hospital in Sasaram, Bihar. Int J Community Med Public Health. 2016. Available from: http://ijcmph.com/index.php/ijcmph/article/view/757.

[15] Barrera R, Amador M, MacKay AJ. Population dynamics of Aedes aegypti and dengue as influenced by weather and human behavior in San Juan, Puerto Rico. Turell MJ, editor. PLoS Negl Trop Dis. 2011;5(12):e1378. 22206021 10.1371/journal.pntd.0001378 PMC3243685

[16] Stoddard ST, Morrison AC, Vazquez-Prokopec GM, Paz Soldan V, Kochel TJ, Kitron U, et al. The role of human movement in the transmission of vector-borne pathogens. Kittayapong P, editor. PLoS Negl Trop Dis. 2009;3(7):e481. 19621090 10.1371/journal.pntd.0000481 PMC2710008

[17] Wilder-Smith A, Gubler DJ. Geographic expansion of dengue: the impact of international travel. Med Clin North Am. 2008;92(6):1377–90. 19061757 10.1016/j.mcna.2008.07.002

[18] Stoddard ST, Forshey BM, Morrison AC, Paz-Soldan VA, Vazquez-Prokopec GM, Astete H, et al. House-to-house human movement drives dengue virus transmission. Proc Natl Acad Sci USA. 2013;110(3):994–9. 23277539 10.1073/pnas.1213349110 PMC3549073

[19] Lai YJ, Lai HH, Chen YY, Ko MC, Chen CC, Chuang PH, et al. Low socio-economic status associated with increased risk of dengue haemorrhagic fever in Taiwanese patients with dengue fever: a population-based cohort study. Trans R Soc Trop Med Hyg. 2019;114(2):115–20. 10.1093/trstmh/trz103 31688926

[20] Marmot M, Friel S, Bell R, Houweling TA, Taylor S. Closing the gap in a generation: health equity through action on the social determinants of health. Lancet. 2008;372(9650):1661–9. 18994664 10.1016/S0140-6736(08)61690-6

[21] Alyousefi TAA, Abdul-Ghani R, Mahdy MAK, Al-Eryani SMA, Al-Mekhlafi AM, Raja YA, et al. A household-based survey of knowledge, attitudes and practices towards dengue fever among local urban communities in Taiz Governorate, Yemen. BMC Infect Dis. 2016;16(1):1–9. 27717333 10.1186/s12879-016-1895-2 PMC5054547

[22] Chandren JR, Wong LP, AbuBakar S. Practices of dengue fever prevention and the associated factors among the Orang Asli in Peninsular Malaysia. PLoS Negl Trop Dis. 2015;9(8):1–17. 10.1371/journal.pntd.0003954 PMC4534093 26267905

[23] Wong LP, AbuBakar S, Chinna K. Community knowledge, health beliefs, practices and experiences related to dengue fever and its association with IgG seropositivity. PLoS Negl Trop Dis. 2014;8(5):e2789. 24853259 10.1371/journal.pntd.0002789 PMC4031145

[24] Espinoza-Gómez F, Hernández-Suárez CMCCR. Educational campaign versus malathion spraying for the control of Aedes aegypti in Colima. Mexico J Epidemiol Community Health. 2002;56(2):148–52. 11812816 10.1136/jech.56.2.148 PMC1732074

[25] Khun S, Manderson L. Community participation and social engagement in the prevention and control of dengue fever in rural. Cambodia. 2008;32:145–55.

[26] Espinoza-Gomez F. Educational campaign versus malathion spraying for the control of Aedes aegypti in Colima, Mexico. J Epidemiol Community Health. 2002;56(2):148–52. 11812816 10.1136/jech.56.2.148 PMC1732074

[27] Zaki R, Roffeei SN, Hii YL, Yahya A, Appannan M, Said MA, et al. Public perception and attitude towards dengue prevention activity and response to dengue early warning in Malaysia. Tambo E, editor. PLoS ONE. 2019;14(2):e0212497. 30818394 10.1371/journal.pone.0212497 PMC6394956

[28] Rahman AA, Zainuddin H, Minhat HS, Juni MH, Mazeli MI. Community perception towards dengue and dengue prevention program among residences of a rural settlement in Jempol, Negeri Sembilan. Int J Public Health Clin Sci. 2014;1(1):13–26.

[29] Wan Rosli WR, Abdul Rahman S, Parhar JK, Suhaimi MI. Positive impact of educational intervention on knowledge, attitude, and practice towards dengue among university students in Malaysia. J Public Health (Berl). 2019;27(4):461–71.

[30] Hossain MJ, Das M, Islam MW, Shahjahan M, Ferdous J. Community engagement and social participation in dengue prevention: a cross-sectional study in Dhaka City. Health Sci Rep. 2024;7(4):e2022. 38572117 10.1002/hsr2.2022 PMC10987789

[31] Dhimal M, Aryal KK, Dhimal ML, Gautam I, Singh SP, Bhusal CL, et al. Knowledge, attitude and practice regarding dengue fever among the healthy population of highland and lowland communities in central Nepal. PLoS ONE. 2014;9(7):e102028. 25007284 10.1371/journal.pone.0102028 PMC4090170

[32] Description of the local level. Available from: https://sthaniya.gov.np/gis/.

[33] Brief Introduction |. Lalitpur Metropolitan City. https://lalitpurmun.gov.np/node/3. Cited 2024 Sep 11.

[34] National Statistics Office. National population and housing census 2021. Volume 01: National report. Reprint. Thapathali, Kathmandu: National Statistics Office; 2023. 598 p.

[35] Maharjan M, Aryal A, Man Shakya B, Talchabhadel R, Thapa BR, Kumar S. Evaluation of urban heat island (UHI) using satellite images in densely populated cities of South Asia. Earth. 2021;2(1):86–110.

[36] Epidemiology and Disease Control Division. Situation update of dengue 2022 (As of 31st Dec, 2022). 2022.

[37] Creswell JWL, Clark VP. Choosing a mixed methods design. Designing Conducting Mixed Methods Res. 2010;2(1):53–106.

[38] Creswell JW, Clark VLP. Designing and conducting mixed methods research. Los Angeles: SAGE Publications; 2018. p 2017.

[39] Bluman AG. Elementary statistics: a step by step approach. Ninth edition. New York, NY: McGraw-Hill Education; 2014. p. 1.

[40] Bluman AG. Elementary statistics: a step by step approach. 9th ed. New York: McGraw-Hill Education; 2014.

[41] Martin N, Marshall. Sampling for qualitative research. Fam Pract. 1996;13(6):522–55. 9023528 10.1093/fampra/13.6.522

[42] Malterud K, Siersma VD, Guassora AD. Sample size in qualitative interview studies: guided by information power. Qual Health Res. 2016;26(13):1753–60. 26613970 10.1177/1049732315617444

[43] Joshi SK, Acharya K. Modification of Kuppuswamy’s socioeconomic status scale in the context of Nepal, 2019. Kathmandu Univ Med J. 2019;17(65):1–2. 31734669

[44] Phuyal P, Kramer IM, Kuch U, Magdeburg A, Groneberg DA, Lamichhane Dhimal M, et al. The knowledge, attitude and practice of community people on dengue fever in central Nepal: a cross-sectional study. BMC Infect Dis. 2022;22(1):1–18. 35549884 10.1186/s12879-022-07404-4 PMC9096776

[45] Dhimal M, Aryal KK, Dhimal ML, Gautam I, Singh SP, Bhusal CL, et al. Knowledge, attitude and practice regarding dengue fever among the healthy population of highland and lowland communities in central Nepal. Halsey ES, editor. PLoS ONE. 2014;9(7):e102028. 25007284 10.1371/journal.pone.0102028 PMC4090170

[46] Harapan H, Rajamoorthy Y, Anwar S, Bustamam A, Radiansyah A, Angraini P, et al. Knowledge, attitude, and practice regarding dengue virus infection among inhabitants of Aceh, Indonesia: a cross-sectional study. BMC Infect Dis. 2018;18(1):96. 29486714 10.1186/s12879-018-3006-z PMC5830327

[47] Selvarajoo S, Liew JWK, Tan W, Lim XY, Refai WF, Zaki RA, et al. Knowledge, attitude and practice on dengue prevention and dengue seroprevalence in a dengue hotspot in Malaysia: a cross-sectional study. Sci Rep. 2020;10(1):9534. 32533017 10.1038/s41598-020-66212-5 PMC7293214

[48] Zhang Z. Model building strategy for logistic regression: purposeful selection. Annals Translational Med. 2016;4(6):4–10. 10.21037/atm.2016.02.15 PMC4828741 27127764

[49] Bendel RB, Afifi AA. Comparison of stopping rules in forward “stepwise” regression. J Am Stat Assoc. 1977;72(357):46–53.

[50] Hosmer DW, Lemeshow S. Applied Logistic Regression.pdf. 2000. p. 161–4.

[51] Khadka RB, Neupane B, Chaudhary GP, Karki K, Pokhrel AP, Pant D, et al. Dengue: rapid diagnostic testing in a tertiary care setting in Butwal, Nepal. J Pure Appl Microbiol. 2023;17(1):380–4.

[52] Paudel AK, Chhetri MR, Devkota N, Maharjan PL, Pokhrel S, Kaphle M. Knowledge and practices on dengue prevention among the people of Buddhabhumi Municipality of Nepal: a cross-sectional study. Nepal Med Coll J. 2023;25(1):42–8.

[53] Paudel D, Kakchapati S, Lageju N, Karki S, Dhungana J, Regmi S, et al. Factors influencing the knowledge, attitude, and practices of police personnel toward dengue fever in Kathmandu, Nepal. J Occup Health. 2023;65(1):1–12. 10.1002/1348-9585.12421 PMC10476170 37664983

[54] Neupane B, Rijal KR, Banjara MR. Knowledge and prevention measures against dengue in southern Nepal. J Coastal Life Med. 2014;2(12):998–1001.

[55] Harapan H, Rajamoorthy Y, Anwar S, Bustamam A, Radiansyah A, Angraini P, et al. Knowledge, attitude, and practice regarding dengue virus infection among inhabitants of Aceh, Indonesia: a cross-sectional study. BMC Infect Dis. 2018;18(1):1–16. 29486714 10.1186/s12879-018-3006-z PMC5830327

[56] Shuaib F, Todd D, Campbell-Stennett D, Ehiri J, Jolly PE. Knowledge, attitudes and practices regarding dengue infection in Westmoreland, Jamaica. The West Indian Medical Journal. 2010;59(2):139–46. 21132094 PMC2996104

[57] Kumar A, Rajendran R, Tewari SC, Arunachalam N, Ayanar K, Krishnamoorthi R, et al. Studies on community knowledge and behavior following a dengue epidemic in Chennai city, Tamil Nadu. India Trop Biomed. 2010;27(2):330–6. 20962733

[58] Gunasekara T, Velathanthiri V, Weerasekara M, Fernando S, Peelawattage M, Guruge D, et al. Knowledge, attitudes and practices regarding dengue fever in a suburban community in Sri Lanka. Galle Med J. 2012;17(1):4355.

[59] Nagoor K, SB D, Reddy NB, Kahn S, Kalluri RJ, John KR. Knowledge, attitude and practice on dengue fever and its prevention and control measures in urban slums of South India. Int J Community Med Public Health. 2017;4(8):3013.

[60] Jeelani S, Sabesan S, Subramanian S. Community knowledge, awareness and preventive practices regarding dengue fever in Puducherry – South India. Public Health. 2015;129(6):790–6. 25863688 10.1016/j.puhe.2015.02.026

[61] Siddiqui TR, Ghazal S, Bibi S, Ahmed W, Sajjad SF. Use of the health belief model for the assessment of public knowledge and household preventive practices in Karachi, Pakistan, a dengue-endemic city. Rabaa MA, editor. PLoS Negl Trop Dis. 2016;10(11):e0005129. 27832074 10.1371/journal.pntd.0005129 PMC5104346

[62] Sayavong C, Chompikul J, Wongsawass S, Rattanapan C. Knowledge, attitudes and preventive behaviors related to dengue vector breeding control measures among adults in communities of Vientiane, capital of the Lao PDR. J Infect Public Health. 2015;8(5):466–73. 25922218 10.1016/j.jiph.2015.03.005

[63] Al-Dubai SA, Ganasegeran K, Alwan MR, Alshagga MA, Saif-Ali R. Factors affecting dengue fever knowledge, attitudes and practices among selected urban, semi-urban and rural communities in Malaysia. Southeast Asian J Trop Med Public Health. 2013;44(1):37–49. 23682436

[64] Liu H, Xu JW, Ai Z, Yu Y, Yu B. Treatment seeking behavior and associated factors of suspected dengue fever among Shan people in eastern Shan special region IV, Myanmar: a cross-sectional study. BMC Health Serv Res. 2020;20(1):1–7. 10.1186/s12913-020-05163-z PMC7164341 32299436

[65] Verma R, Bhalla K, Dhankar M, Kumar R, Dhaka RAG. Practices and knowledge regarding dengue infection among the rural community of Haryana. J Family Med Prim Care. 2019;8(5):1752–4. 31198749 10.4103/jfmpc.jfmpc_6_19 PMC6559082

[66] Van Benthem BHB, Khantikul N, Panart K, Kessels PJ, Somboon P, Oskam L. Knowledge and use of prevention measures related to dengue in northern Thailand. Trop Med Int Health. 2002;7(11):993–1000. 12390606 10.1046/j.1365-3156.2002.00950.x

